# Sex differences in cancer outcomes across the range of eGFR

**DOI:** 10.1093/ndt/gfae059

**Published:** 2024-03-09

**Authors:** Richard Shemilt, Michael K Sullivan, Peter Hanlon, Bhautesh D Jani, Nicole De La Mata, Brenda Rosales, Benjamin M P Elyan, James A Hedley, Rachel B Cutting, Melanie Wyld, David A McAllister, Angela C Webster, Patrick B Mark, Jennifer S Lees

**Affiliations:** NHS Greater Glasgow and Clyde, G12 0XH, UK; School of Medicine, Dentistry and Nursing, University of Glasgow, Glasgow G12 8QQ, UK; NHS Greater Glasgow and Clyde, G12 0XH, UK; School of Cardiovascular and Metabolic Health, University of Glasgow, Glasgow G12 8TA, UK; School of Health and Wellbeing, University of Glasgow, Glasgow G12 8TB, UK; School of Health and Wellbeing, University of Glasgow, Glasgow G12 8TB, UK; Sydney School of Public Health, University of Sydney, Sydney NSW 2050, Australia; Sydney School of Public Health, University of Sydney, Sydney NSW 2050, Australia; NHS Greater Glasgow and Clyde, G12 0XH, UK; School of Cardiovascular and Metabolic Health, University of Glasgow, Glasgow G12 8TA, UK; Sydney School of Public Health, University of Sydney, Sydney NSW 2050, Australia; Sydney School of Public Health, University of Sydney, Sydney NSW 2050, Australia; Sydney School of Public Health, University of Sydney, Sydney NSW 2050, Australia; School of Health and Wellbeing, University of Glasgow, Glasgow G12 8TB, UK; Sydney School of Public Health, University of Sydney, Sydney NSW 2050, Australia; NHMRC Clinical Trials Centre, University of Sydney, Sydney NSW 2050, Australia; NHS Greater Glasgow and Clyde, G12 0XH, UK; School of Cardiovascular and Metabolic Health, University of Glasgow, Glasgow G12 8TA, UK; NHS Greater Glasgow and Clyde, G12 0XH, UK; School of Cardiovascular and Metabolic Health, University of Glasgow, Glasgow G12 8TA, UK

**Keywords:** cancer, chronic kidney disease, cohort studies, female, male

## Abstract

**Background:**

People with chronic kidney disease (CKD) have increased incidence and mortality of most cancer types. We hypothesized that the odds of presenting with advanced cancer may vary according to differences in estimated glomerular filtration rate (eGFR), that this could contribute to increased all-cause mortality and that sex differences may exist.

**Methods:**

Data were from Secure Anonymised Information Linkage Databank, including people with *de novo* cancer diagnosis (2011–17) and two kidney function tests within 2 years prior to diagnosis to determine baseline eGFR (mL/min/1.73 m^2^). Logistic regression models determined the odds of presenting with advanced cancer by baseline eGFR. Cox proportional hazards models tested associations between baseline eGFR_Cr_ and all-cause mortality.

**Results:**

eGFR <30 was associated with higher odds of presenting with advanced cancer of prostate, breast and female genital organs, but not other cancer sites. Compared with eGFR >75–90, eGFR <30 was associated with greater hazards of all-cause mortality in both sexes, but the association was stronger in females [female: hazard ratio (HR) 1.71, 95% confidence interval (CI) 1.56–1.88; male versus female comparison: HR 0.88, 95% CI 0.78–0.99].

**Conclusions:**

Lower or higher eGFR was not associated with substantially higher odds of presenting with advanced cancer across most cancer sites, but was associated with reduced survival. A stronger association with all-cause mortality in females compared with males with eGFR <30 is concerning and warrants further scrutiny.

## Graphical Abstract

**Graphical Abstract:**
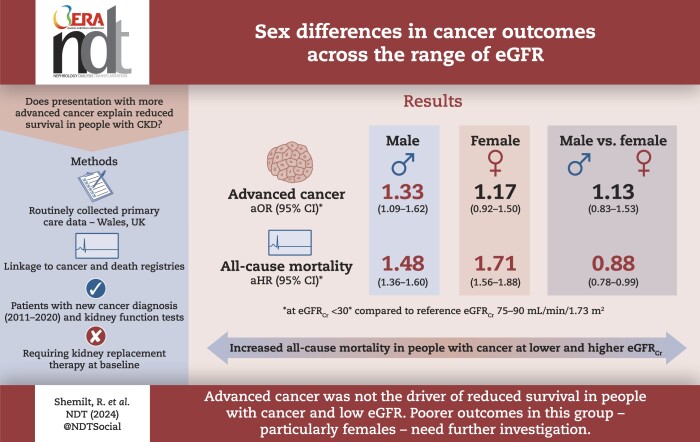

KEY LEARNING POINTS **What was known:**

- Chronic kidney disease (CKD) is associated with increased incidence and reduced survival from most types of cancer.
- In the general population, prognosis is poorer with more advanced cancer and there are well-documented sex differences in cancer incidence and outcomes.
- Previous studies have not investigated the effect of differences in estimated glomerular filtration rate (eGFR) on advanced cancer stage at diagnosis and outcome.

**This study adds:**

- Data from a nationally representative cohort, in which eGFR <45 was associated with increased odds of presenting with advanced cancer of breast and prostate, but not other solid organ cancers.
- All-cause mortality was increased in all participants with eGFR <30 but this association was stronger in females.
- The paradoxical association seen between high eGFR, advanced cancer and all-cause mortality was partially attenuated by adjustment for surrogate markers of syndrome of inappropriate anti-diuretic hormone (SIADH) and frailty.

**Potential impact:**

- Advanced cancer stage at presentation was not the primary driver of poorer cancer outcomes associated with differences in eGFR, which suggests that differences in post-diagnosis cancer care may exist that contribute to reduced survival.
- Scrutiny of the selection, efficacy and safety of cancer treatment in people with reduced eGFR is warranted—particularly in females.

## INTRODUCTION

Chronic kidney disease (CKD) poses a significant healthcare burden globally: it affects approximately 11%–13% of the population [1], and is increasingly common, driven by ageing and multi-morbidity [2]. CKD is more common among people with other comorbid diseases, particularly cardiovascular disease and cancer. Depending on cancer site, CKD may be present in up to 50% of people diagnosed with cancer [3]. There are sex differences in cancer outcomes: in the general population, females have better survival from cancer than males; however, females with CKD and kidney failure have worse relative survival, more excess deaths and more years of life lost to cancer [4, 5]. The loss of female survival advantage in CKD is not well understood.

With increasing severity of CKD [reduced estimated glomerular filtration rate (eGFR) and albuminuria], the risk of cancer death rises [6–8], although the mechanisms are uncertain. In the general population, diagnosis of cancer at a more advanced stage results in poorer outcomes [9, 10]: curative treatment options are limited with advanced staging [9, 11]. The presence of CKD may further restrict access to, and safety and/or efficacy of cancer treatments, including surgery and systemic anti-cancer therapies (SACT) [12, 13]. However, CKD may also influence presenting cancer stage: there may be differences in cancer biology, in the timing and nature of healthcare interactions, and/or in the investigation and/or management of non-specific symptoms seen commonly in both CKD and cancer (e.g. anaemia and weight loss). It is conceivable that presentation with more advanced cancer stage explains reduced survival after a cancer diagnosis among people with CKD; this has not previously been investigated.

In the general population, sex differences in cancer incidence and outcome are well-documented [14]. Predominant cancer sites and associated prognosis vary considerably in people of male and female sex, with a significant impact on overall sex differences in cancer outcomes [15]. In some cases this is due to obvious anatomical, hormonal or epidemiological differences. Less is known about other factors which may influence differences in cancer outcome between sexes—such as timing of presentation and variation in treatment strategy.

Using data from a large primary care cohort, we sought to address our hypotheses that differences in kidney function—measured by eGFR—increase likelihood of presentation with advanced cancer, that more invasive cancer stage at presentation is associated with reduced survival in people with low eGFR, and that sex differences exist in cancer presentation and survival.

## MATERIALS AND METHODS

### Data sources and population

Data were from the Secure Anonymised Information Linkage Databank (SAIL), a Welsh primary care database with linkage to cancer (Wales Cancer Intelligence and Surveillance Unit) and death (Office for National Statistics) registries, in a setting where universal healthcare is available through the National Health Service (NHS). Participants were included if they had: (i) a *de novo* diagnosis of malignant cancer between 1 January 2011 and 31 December 2017 [by International Classification of Diseases, Tenth Revision (ICD-10) code C00–C75 (excluding C44—reporting of non-melanoma skin cancers are not mandated in cancer registries)]; and (ii) if they had kidney function tested at least twice, at least 3 months apart, and within 2 years prior to the cancer diagnosis. Participants receiving maintenance kidney replacement therapy (KRT; dialysis or a kidney transplant) at the time of cancer diagnosis were excluded.

eGFR was calculated from serum creatinine without including the race coefficient (eGFR_Cr_; CKD Epidemiology Collaboration 2009 equation [16]); the method currently recommended for use in UK populations [17]. In keeping with many primary care populations, albuminuria was not consistently available for CKD staging [18].

Participant demographics were extracted from the primary care record. Age was calculated in years between date of birth and date of first cancer diagnosis. Sex was recorded in the clinical record as ‘male’ or ‘female’. Smoking status was coded as ‘never smoker’, ‘ex-smoker’ or ‘current smoker’. Comorbidites were defined according to a previously published list of 40 long-term conditions [19], defined using Read Codes from primary care records as previously described [20, 21]. Comorbidity count was calculated as the sum of long-term conditions, excluding CKD and cancer. Deprivation status was expressed using the Welsh Index of Multiple Deprivation (WIMD) 2011 [22], which considers eight weighted indices (income, employment, health, education, geographical access to services, housing, physical environment and community safety) according to home postcode to provide a ranked WIMD score. WIMD was expressed in deciles from 1 (most deprived) to 10 (least deprived).

For site-specific analyses, cancer site was determined from ICD-10 codes as the first cancer in the follow-up period. A full list of groupings by cancer site is available in Table 1. For site-specific analyses, we excluded cancers where there were fewer than 500 diagnoses in the total population for reasons of patient confidentiality, and to avoid invalid statistical inference. This excluded people with a first cancer of the male genital organs, bone, thyroid, adrenal, endocrine and brain/central nervous system cancers from further analysis.

**Table 1:** Baseline data.

	All	Female	Male	*P*-value
*N* (%)	66 128 (100)	30 857 (46.7)	35 271 (53.3)	
Age (years), mean (SD)	69.9 (12.5)	69.1 (13.8)	70.6 (11.1)	<.001
eGFR (mL/min/1.73 m^2^), median (IQR)	78.5 (63.0–89.8)	78.3 (62.9–90.0)	78.5 (63.2–89.7)	.543
eGFR category (mL/min/1.73 m^2^), *n* (%)				<.001
eGFR >120	21 026 (31.8)	9630 (31.2)	11 396 (32.3)	
eGFR 105–<120	283 (0.4)	152 (0.5)	131 (0.4)	
eGFR 90–<105	2290 (3.5)	1234 (4.0)	1056 (3.0)	
eGFR 75–<90	13 680 (20.7)	6327 (20.5)	7353 (20.8)	
eGFR 60–<75	14 641 (22.1)	6849 (22.2)	7792 (22.1)	
eGFR 45–<60	8069 (12.2)	3795 (12.3)	4274 (12.1)	
eGFR 30–<45	4428 (6.7)	2133 (6.9)	2295 (6.5)	
eGFR <30	1711 (2.6)	737 (2.4)	974 (2.8)	
Smoking status, *n* (%)				<.001
Current smoker	13 729 (20.8)	6426 (20.8)	7303 (20.7)	
Ex-smoker	19 847 (30.0)	7244 (23.5)	12 603 (35.7)	
Non-smoker	21 348 (32.3)	11 438 (37.1)	9910 (28.1)	
Missing	11 204 (16.9)	5749 (18.6)	5455 (15.5)	
Comorbidity count, median (IQR)	2 (1–4)	2 (1–4)	2 (1–4)	<.001
WIMD decile, median (IQR)	5 (3–8)	5 (3–8)	6 (3–8)	<.001
Cancer stage, *n* (%)				<.001
1 (least advanced)	13 277 (20.1)	8162 (26.5)	5115 (14.5)	
2	13 118 (19.8)	5663 (18.4)	7455 (21.1)	
3	10 900 (16.5)	4703 (15.2)	6197 (17.6)	
4 (most advanced)	15 371 (23.2)	6089 (19.7)	9282 (26.3)	
Unknown	13 462 (20.4)	6240 (20.2)	7222 (20.5)	

### Outcomes

We were interested in the following outcomes:

- presentation with advanced cancer; i.e. stage 3 or 4 cancer by Tumour Node Metastases (TNM), numeric grading systems, or—for female genital organ cancers—International Federation of Gynecology and Obstetrics;
- death (from any cause) after cancer diagnosis during the follow-up period.

### Statistical analysis

Data summaries are stratified by sex and expressed as mean (standard deviation, SD), median (interquartile range, IQR) and count (%), and compared using *t*-test, Kruskal–Wallis and chi-squared test as appropriate.

To determine odds of presenting with advanced cancer by eGFR (overall, and by cancer site), we applied logistic regression models, adjusted for age, deprivation status, smoking status, comorbidity count plus cancer site (for overall, but not site-specific models). Where staging information was unavailable (where stage was recorded as ‘GX’ or where no staging information was recorded within the cancer registry) for other solid organ cancers, presenting cancer stage was allocated as ‘unknown’.

To determine hazards of death (from any cause; overall, and by cancer site) after cancer diagnosis by eGFR, we constructed Cox proportional hazards models adjusted for age, deprivation status, smoking status, comorbidity count, cancer site (for overall, but not site-specific models) and cancer stage at presentation. Follow-up was from cancer diagnosis until the sooner of date of death or 1 October 2020.

To describe the potential associations across a range of kidney function, eGFR was categorized in 15 mL/min/1.73 m^2^ decrements (using the two most recent eGFR measurements, taken at least 3 months apart and within 2 years prior to cancer diagnosis) as follows: eGFR >120, >105–120, >90–105, >75–90 (reference), >60–75, >45–60, >30–45, <30. Where there were smaller numbers at eGFR extremes (e.g. testing associations for site-specific cancers), the top and bottom two categories were collapsed. eGFR categories for site-specific cancers were therefore as follows: eGFR >105, >90–105, >75–90 (reference), >60–75, >45–60, ≤45.

In exploratory analyses, we tested the potential role of other indicators of disease severity—the syndrome of inappropriate anti-diuretic hormone (SIADH; considering serum urea, sodium and uric acid as potential surrogate markers [23]) and/or unmeasured frailty characteristics (using serum albumin as a potential surrogate [24])—on the association between eGFR, presentation with advanced cancer and all-cause mortality. We extracted values within 2 years prior to the cancer diagnosis, and selected the single value closest to the diagnosis of cancer. We assessed the distribution of age, urea, creatinine, sodium and albumin by diagnosis of advanced cancer, sex and baseline eGFR. We did not include urate due to very high levels of missingness. We tested whether inclusion of urea, sodium and albumin in logistic regression and survival models altered the relationship between eGFR and diagnosis of advanced cancer or all-cause mortality.

Evidence of a statistical interaction was sought between sex and eGFR category in both logistic regression and Cox proportional hazards models (interaction *P* < .001 was considered significant). Results are presented: (i) stratified by sex and (ii) indicating where significant interactions exist between sex and eGFR. In sex-stratified analyses for cancer survival, we additionally tested for an interaction between age and eGFR, in this case considering age as a continuous variable (per 10 mL/min/1.73 m^2^ decrease below or increase above the reference group of 75–90 mL/min/1.73 m^2^) to avoid multiple significance testing across eGFR categories. To account for a substantial proportion of included patients who had missing cancer stage, sensitivity analyses (assuming highest or lowest possible stage) were conducted.

Analyses were conducted using *tidyverse, nephro, broom, tableone* and *survival* packages for R statistical software (version 4.1.3).

## RESULTS

Of 141 784 patients with a diagnosis of cancer, there were 66 128 with two available kidney function measures who were included in the analyses. People with cancer who were excluded due to insufficient kidney function tests to meet eligibility criteria were younger, with similar median eGFR_Cr_ (based on single measure alone), comorbidity count and deprivation status, and similar proportions of females and current smokers (Supplementary data, Table S1).

In our included cohort, 46.7% were female, with mean age in females 69.1 (SD 13.8) years and in males 70.6 (SD 11.1) years (Table 1). There were 14 208 individuals (21.5% overall; 21.6% in females and 21.4% in males) with eGFR <60 mL/min/1.73m^2^ at baseline. Over median follow-up time 3.1 (IQR 0.5–5.7) years in females and 2.9 (IQR 0.5–5.5) years in males, there were 17 303 deaths in females and 20 855 deaths in males. Median survival times for site-specific cancers were shortest for abdominal and respiratory cancers, and longest for melanoma in both males and females (Table 2).

**Table 2:** Overall and site-specific cancer diagnoses, deaths and survival times in females and males.

Cancer site	ICD-10 code	Sex	*N*	*N* deaths (%)	Survival time, median (IQR)
All sites	C00–75 (excluding C44)	Female	30 857	17 303 (56.1)	3.1 (0.5–5.7)
		Male	35 271	20 855 (59.1)	2.9 (0.5–5.5)
Abdominal	C22–26	Female	2095	1953 (93.2)	0.3 (0.1–0.9)
		Male	2342	2193 (93.6)	0.3 (0.1–1.1)
Digestive tract	C15–21	Female	5644	3731 (66.1)	1.8 (0.4–5.0)
		Male	8123	5539 (68.2)	1.9 (0.4–4.8)
Head and neck	C00–14, C30–32	Female	696	344 (49.4)	3.5 (1.1–5.9)
		Male	1748	898 (51.4)	3.6 (1.3–5.9)
Lung	C33–34	Female	4946	4423 (89.4)	0.5 (0.1–1.7)
		Male	5482	5016 (91.5)	0.4 (0.1–1.2)
Melanoma	C43	Female	1266	330 (26.1)	5.2 (3.3–7.2)
		Male	1317	448 (34)	4.5 (3.0–6.6)
Other	C37–38, C45–49, C69–72	Female	912	677 (74.2)	1.0 (0.2–3.8)
		Male	1433	1170 (81.6)	0.8 (0.2–2.9)
Renal tract	C64–67	Female	1432	935 (65.3)	1.9 (0.4–5.2)
		Male	2733	1749 (64)	2.6 (0.6–5.4)
Breast	C50	Female	8954	2656 (29.7)	5.0 (3.2–7.0)
Female genital tract	C51–58	Female	4522	2171 (48)	3.7 (1.2–6.3)
Prostate	C61	Male	11 470	3642 (31.8)	4.7 (3.2–6.8)

### Comparison of males versus females without accounting for kidney function

Adjusted for age alone, males were more likely than females to present with advanced cancer [odds ratio (OR) 1.51, 95% confidence interval (CI) 1.47–1.57; *P* < .001]. The association was partially attenuated in fully adjusted models (OR 1.15, 95% CI 1.09–1.22; *P* < .001). Adjusted for age alone, males had similar hazards of death compared with females [hazard ratio (HR) 1.01, 95% CI 0.99–1.03; *P* = .48]; however, in fully adjusted models (adjusted for cancer site and stage at presentation but not for eGFR category), males had higher hazards of all-cause mortality after a cancer diagnosis than females (HR 1.11, 95% CI 1.08–1.13; *P* < .001). Without accounting for kidney function, females had a survival advantage compared with males.

### Risk of presenting with advanced cancer across the range of eGFR

**Figure 1:**
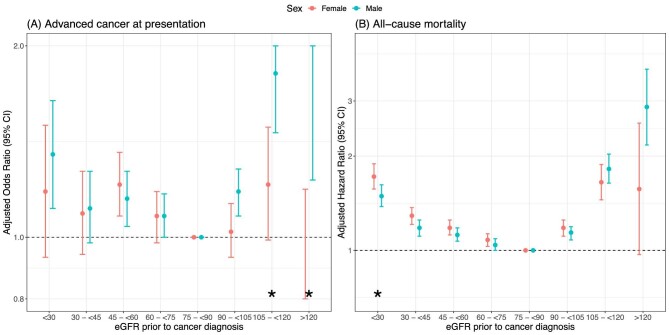
(**A**) Plot displaying OR (95% CI) of presentation with advanced cancer, adjusted for age, smoking status, deprivation status, number of comorbidities and cancer site. (**B**) Plot displaying HR (95% CI) of death after cancer diagnosis, adjusted for age, smoking status, deprivation status, number of comorbidities, cancer site and presenting cancer stage. Results are stratified by sex. Asterisk indicates presence of a significant interaction between sex and eGFR category. Reference eGFR category: 75–<90 mL/min/1.73 m^2^.

In both males and females, there were small increased odds of presenting with advanced cancer when all sites were included in people with extremes of eGFR (Supplementary data, Table S2 and Fig. 1A). This was most pronounced at very low eGFR (<30) and very high eGFR (>105–120; >120) in males. In females, OR for presenting with advanced cancer with low eGFR (<30; 30–<45) and high eGFR (>105–120; >120) crossed the null. There was a statistical interaction between eGFR and sex at high eGFR: males were at higher odds of presenting with advanced cancer; Fig. 1A).

**Figure 2:**
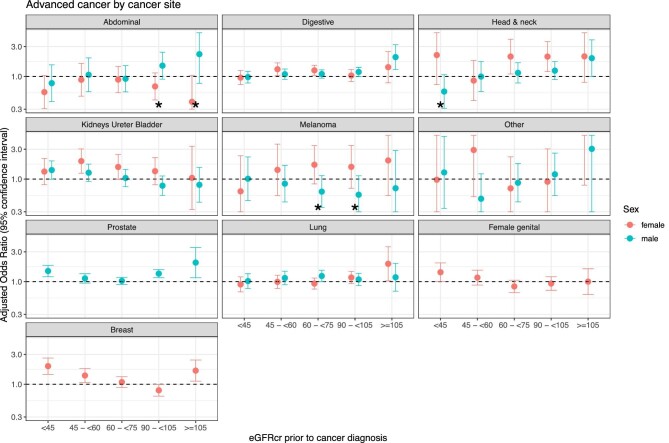
Plot displaying OR (95% CI) of presentation with advanced (stage 3 or 4) site-specific cancer. Models are adjusted for age, sex, smoking status, deprivation status and number of comorbidities. Results are stratified by sex and cancer site. Asterisk indicates presence of a significant interaction between sex and eGFR category.

The likelihood of presenting with advanced site-specific cancer differed by sex and eGFR (Supplementary data, Table S3 and Fig. 2). Females with eGFR <45 were more likely to present with advanced breast and female genital tract cancers than the reference group (eGFR >75–90). A similar finding was seen in males with prostate cancer. There was no significant association between lower eGFR and likelihood of presenting with advanced cancer across any other solid organ cancer site. Very high eGFR (≥105) was associated with increased likelihood of presenting with advanced breast cancers (in females), prostate cancers (in males), digestive tract cancers (in males) and lung cancers (in females).

### All-cause mortality after cancer diagnosis across the range of eGFR

In males and females, adjusted hazards of death after a cancer diagnosis were higher with eGFR both lower (<75) and higher (≥90) than the reference category; the pattern was more pronounced at extremes of eGFR (Fig. 1B; Supplementary data, Table S4). There was a statistical interaction between eGFR and sex at eGFR <30 (males had lower hazards of cancer death than females: HR 0.88, 95% CI 0.78–0.99; *P* = .04) and at eGFR >120 (males had higher hazards of cancer death than females: HR 1.80, 95% CI 1.02–3.16; *P* = .04; Supplementary data, Table S4 and Fig. 1B). On sensitivity analyses, assuming the maximum and minimum possible cancer stage for those with missing information, findings were similar (data available on request). In sex-stratified analyses, we further identified a significant interaction between eGFR and age in both male and female participants. There was a stronger association between eGFR that was lower and higher than the reference group in younger individuals (Supplementary data, Table S5).

**Figure 3:**
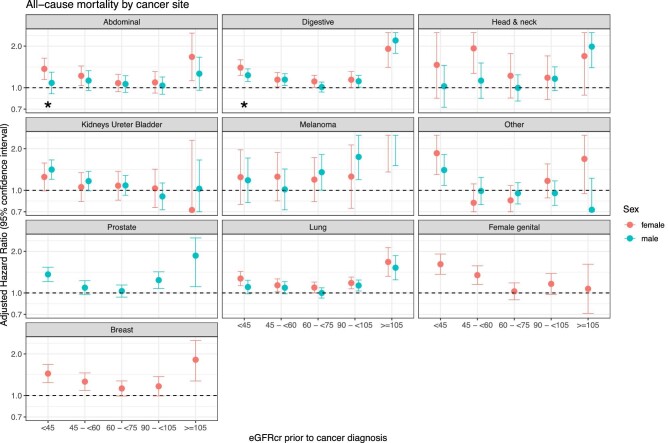
Plot displaying HR (95% CI) of death (any cause) after site-specific cancer diagnosis. Models are adjusted for age, sex, smoking status, deprivation status, number of comorbidities and presenting cancer stage. Results are stratified by sex and cancer site. Asterisk indicates presence of a significant interaction between sex and eGFR category.

In site-specific cancers, eGFR <45 was associated with higher hazards of death in people diagnosed with abdominal organ cancers (females more than males) and digestive tract cancers (females more than males), with similar higher hazards in males and females for haematological cancers including myeloma, renal tract, lung and non-melanoma skin cancers, prostate (males only) and breast (females only) cancers (Supplementary data, Table S6 and Fig. 3). eGFR ≥105 was associated with higher hazards of death in both males and females with abdominal organ, digestive tract, head and neck, melanoma, non-melanoma skin, lung, breast (females only) and prostate (males only) cancers (Fig. 3).

### Exploratory analyses considering markers of SIADH and frailty

Patients with higher eGFR ≥100 were younger, with lower urea and creatinine, but similar sodium and albumin values, compared with those with eGFR <100. The distribution of urea, creatinine, sodium and albumin were slightly skewed towards lower values in patients presenting with advanced cancer (Supplementary data, Fig. S1). Inclusion of urea, sodium and albumin in the logistic regression models partially attenuated the relationship between very high eGFR and higher likelihood of presenting with advanced cancer in males, and the interaction between sex*eGFR in the highest eGFR categories was lost. Inclusion of sodium, urea and albumin values in Cox proportional hazards models partially attenuated the association between high eGFR and all-cause mortality seen previously in males, and there was no longer a significant interaction between sex and high eGFR in the highest eGFR category. However, the addition of these variables enhanced the sex differences in the association between low eGFR and mortality: females with eGFR <30 and 30–<45 mL/min/1.73 m^2^ had higher relative hazards of death than males (Supplementary data, Fig. S2).

## DISCUSSION

We are not aware of any prior studies that have examined sex differences in presenting cancer stage and how this may be affected by differences in eGFR. We found that males with very low and very high eGFR were more likely to present with advanced cancer—a finding that was partially attenuated by accounting for biochemical surrogate markers of SIADH or frailty. Our study is in keeping with several prior analyses that show higher hazards of death associated with cancer in people with lower eGFR but also shows poorer outcomes associated with higher eGFR [4, 6–8, 25]. Our data suggest that in people with lower and higher eGFR, there are notable sex differences in outcomes post-cancer diagnosis across several cancer sites. One prior study has identified that CKD is associated with more years of life lost to cancer in females than in males [4]. To our knowledge, ours is the first study to report that sex differences in people with lower and higher eGFR still exist after accounting for both the cancer site and stage at presentation.

### Cancer survival difference across the range of eGFR

Differences in cancer survival between populations with and without extremes of eGFR suggest that differences in post-diagnosis care may exist. Due to widespread exclusion from trials of SACT [26, 27], there is a paucity of evidence of the efficacy and safety of SACT among people with the extremes of eGFR [27]. However, most anti-cancer drugs are administered near the maximum tolerated dose and have a narrow therapeutic index [28]. Dosing considerations (and kidney function) are therefore particularly important.

Cytotoxic agents including platinum-based chemotherapy (such as carboplatin) and alkylating agents (such as ifosfamide) may cause a number of renal complications including acute tubular injury leading to chronic tubulointerstitial fibrosis [29]. Lower baseline eGFR makes these complications more likely and their consequences potentially more serious, meaning that these agents are often avoided altogether in this group [30].

Improved cancer outcomes in the general population have been achieved through targeting specific immune mediators and avoiding many of the toxic systemic effects of cytotoxic chemotherapies [13]. Many targeted agents primarily undergo hepatic metabolism: no dose adjustment is expected even in advanced CKD [28] and there is case-series evidence of SACT being given safely to patients on KRT [31]. Improvements in cancer survival seen in the general population have not been matched in people with CKD, and it is unclear (i) to what extent newer SACT are used in people across the disease spectrum of CKD, and (ii) whether SACT efficacy and safety profiles are similar in CKD to those in the general population [30]. Given that CKD and cancer often co-exist, a better understanding of SACT use in CKD (both in trials and in the post-licensing period) is essential to improve the provision of evidence-based cancer care to people with CKD.

### Sex differences in cancer survival

Differences in treatment selection, efficacy and safety profiles may explain reduced relative survival in female compared with male participants with lower eGFR.

In the studied population, where universal healthcare is available through the NHS, sex differences in cancer survival should not, in principle, reflect differential availability of cancer treatments. Treatment selection may vary by sex and could be affected both by clinical judgement and patient preference. We are not aware of any prior studies of sex differences in patient preference for cancer treatments, particularly in the context of kidney disease, but it is plausible that differences in gender roles, health-related behaviours and attitude to risk could result in sex differences in cancer treatment selection. We are not aware of any routine healthcare data system where this information is captured.

It is plausible that there are sex differences in cancer treatment efficacy and safety. A recent meta-analysis of sex differences in cancer immunotherapy efficacy showed a significantly greater relative reduction in risk of death in males treated with immunotherapy compared with females [32]. This analysis also highlighted disparities in the current evidence base for cancer therapies in males and females, with males comprising two-thirds of included participants in the 20 randomized controlled trials. Though the inclusion of females in trials has increased since the 1993 reversal of previous Food and Drug Administration guidelines banning females of child-bearing potential from participation in clinical research, male participants still predominate [33]. There is a growing case that trial evidence should be interpreted and applied to clinical practice after taking sex into account. This suggests that review of the efficacy and safety profiles of cancer therapies in females (particularly females with very low eGFR) is particularly urgent.

Beyond differences in treatment, potential reasons for sex differences in cancer outcomes include differences in environmental exposures, gene expression, immunity and hormones [34]. The effects of hormones on innate and adaptive immune responses are increasingly recognized, with oestradiol thought to enhance both cell-mediated and humoral immune responses [35]. This has been postulated to be one factor contributing to the increased incidence of autoimmune disease in females and cancer in males. This is of particular interest in the context of kidney disease and cancer: advancing kidney disease impairs function of the hypothalamic–pituitary–gonadal axis and results in failure of oestradiol levels to peak normally mid-menstrual cycle [36]. Low oestradiol has been associated with worsening kidney function [36]. While the specific mechanisms remain unclear, oestradiol may have an immunomodulatory effect which could contribute to sex differences in cancer incidence, outcome and response to treatment. Though beyond the scope of this study, further investigation is required to understand whether differences in cancer biology underpin poorer cancer outcome in females (compared with males) with low eGFR.

### Non-linear relationship between eGFR_Cr_, advanced cancer stage at diagnosis and survival

There was a notable ‘J-shaped’ relationship between eGFR_Cr_ and advanced cancer stage at diagnosis (in males) and hazards of death after cancer diagnosis. Consistent with findings in other populations, eGFR_Cr_ >90 mL/min/1.73 m^2^ was also associated with poorer survival [8, 37, 38]. However, eGFR calculated using an alternative marker of kidney function (cystatin C—not routinely tested or available for comparison in this population) shows a more biologically plausible, linear association between eGFR and cancer death [8], suggesting that the J-shaped relationship with eGFR_Cr_ reflects flaws in creatinine-based estimation of kidney function. Muscle mass contributes to systemic error in estimation of eGFR_Cr_ which is most significant at extremes, particularly in patients with cachexia, sarcopenia and high muscularity [39]. Patients at higher extremes of eGFR_Cr_ may actually reflect worse kidney function, where low muscle mass results in overestimation of eGFR_Cr_, when in fact low body weight or sarcopenia (common in people with more advanced cancer) may place them in a higher risk group for treatment toxicity and poor outcome [40]. SIADH and unmeasured frailty are further plausible explanations particularly in patients with cancer. Inclusion of surrogate markers of SIADH and frailty partially attenuate the relationship between high eGFR, advanced cancer and all-cause mortality in males, and enhanced the relationship between lower eGFR and mortality in females. These findings strengthen the hypothesis that some of the associations seen, particularly at higher eGFR, are driven by determinants of serum creatinine that are unrelated to kidney function. In this situation, the association between higher eGFR_Cr_ and worse outcome may reflect reverse causality.

### Strengths and limitations

The strengths of this study lie in the capture of nationally representative data in people diagnosed with cancer, using cancer registry data to confirm cancer diagnoses, and biochemical confirmation of eGFR category (rather than clinical coding of CKD). We acknowledge several limitations. First, we have included only patients who had at least two measures of kidney function in advance of cancer diagnosis, disproportionately collecting information on people with reasons to seek regular medical attention. This represents approximately half of all people diagnosed with cancer over the same time period; however, this is a robust method for establishing baseline eGFR [17]. Kidney function is commonly tested in community populations, and especially in older people, those with long-term conditions or in those with symptoms that might be in keeping with cancer. Our selected population was slightly older are more likely to be current smokers than the unselected group, but with similar eGFR_Cr_, number of comorbidities and deprivation status. Our approach has likely captured a substantial proportion of the kidney disease population. Second, we considered any creatinine value available through the primary care record as suitable for inclusion. These values were predominantly collected in the outpatient setting, and are therefore more likely to represent true baseline eGFR, but it is possible that some creatinine values were captured during inpatient hospital episodes. Third, certain types of cancer (such as renal tract cancers and myeloma) may cause impairment of kidney function and reduced eGFR, introducing the possibility of reverse causality. However, our findings were also preserved across a variety of cancer sites in which reverse causality is implausible. Fourth, this study was not designed to explore the role of SIADH or frailty as confounders to the association between eGFR, presentation with advanced cancer and survival. The findings should be considered hypothesis generating. Fifth, we did not have access to detailed screening information (relevant for colorectal, breast and cervical cancer in this population), and cannot comment on the role of screening on sex differences in the diagnosis of advanced cancer or survival in this population. Finally, in keeping with challenges seen in national registries worldwide, cancer stage was unknown for around 20% of participants. Missing stage may have biased the potential association between likelihood of presenting with advanced cancer in either direction. However, findings were similar whether investigating this association in those with complete information and on sensitivity analyses assuming highest or lowest possible stage.

### What next?

Existing data may provide valuable information on the utility of cancer treatments in female and male patients with across the spectrum of eGFR. Trial data may be limited by lack of representativeness; routinely collected data may be limited by confounding by indication. We propose detailed exploration of trial and linked routinely collected data cancer treatment in female and male patients across differences in eGFR. The aims should be to understand if, how and why differences in eGFR affect:

- cancer treatment selection: operative management, radiotherapy, SACT, conservative management;
- treatment delivery: time-to-treatment, dose, duration;
- efficacy: progression-free survival, overall survival;
- safety: serious adverse events, hospitalizations;
- clinical trial enrolment.

### Conclusions

Lower eGFR was associated with higher odds of presenting with advanced cancer of the breast, prostate and female genital organs, but not with advanced cancer of other sites. Higher eGFR was associated with increased odds of presenting with advanced cancer overall in males, and for breast cancer in females. Extremes of eGFR (both higher and lower) are associated with reduced survival in people diagnosed with cancer. The paradoxical association between high eGFR, advanced cancer stage at diagnosis and survival is likely explained by determinants of serum creatinine that are not related to kidney function. Despite an initial survival advantage compared with males, females with eGFR <30 had disproportionately higher hazards of death. Lack of evidence and guidance for cancer treatment in people with CKD may underpin these findings, and augment sex differences. Particularly in cancer types where sex discrepancies exist (abdominal organ and digestive tract cancers), scrutiny of the selection, delivery, efficacy and safety of cancer treatment in people with lower eGFR is warranted.

## Supplementary Material

gfae059_Supplemental_File

## Data Availability

Data are publicly available on application to Secure Anonymised Information Linkage Wales (SAIL; https://saildatabank.com). The current analysis was conducted under project 1214. Model outputs and analysis code will be made available at time of publication (https://github.com/UoGSCMHDataScience/sail_cancer_ckd_public/).
