# Supplementary material for: Sex differences in cancer outcomes across the range of eGFR
Source: Nephrol Dial Transplant. 2024 Mar 9;39(11):1799–808. doi: 10.1093/ndt/gfae059 (PMC11648947; doi:10.1093/ndt/gfae059)
Supplement: gfae059_Supplemental_File [file gfae059_Supplemental_File.pdf]

Supplementary data

Table S1 - Baseline characteristics for patients diagnosed with cancer during follow-up, but who did not have two or more eGFR measures available in the two years prior to diagnosis.

	Female	Male
**N (%)**	34,813 (47.7)	38,164 (52.3)
**Age (years): mean (SD)**	65.0 (14.1)	66.6 (11.5)
**eGFR (mL/min/1.73m^2): median [IQR]**	77.5 [65.1, 90.2]	78.1 [65.6, 90.0]
**Smoking status (%)**		
**Current smoker**	7,294 (21.0)	7,943 (20.8)
**Ex-smoker**	7,965 (22.9)	13,260 (34.7)
**Non-smoker**	12,850 (36.9)	11,022 (28.9)
**Missing**	6,704 (19.3)	5,939 (15.6)
**Comorbidity count: median [IQR]**	2 [1, 4]	2 [1, 4]
**WIMD decile: median [IQR]**	5 [3, 8]	6 [3, 8]

Baseline characteristics for patients diagnosed with cancer during follow-up, but who did not have two or more eGFR measures available in the two years prior to diagnosis.

Table S2 - Odds of presenting with advanced cancer across all sites by eGFR category.

	Female		Male		Male versus female	
**eGFR category (mL/min/1.73m^2)**	**OR (95% CI)**	**P value**	**OR (95% CI)**	**P value**	**OR (95% CI)**	**P value**
**P for trend**		Linear 0.06<br>Non-linear 0.42		Linear 0.089<br>Non-linear <0.001		Linear 0.08<br>Non-linear 0.67
**>120**	0.65 (0.35-1.18)	0.15	2.40 (1.26-4.58)	0.008	3.27 (1.41-7.97)	0.01
**105 - <120**	1.21 (0.98-1.48)	0.08	1.80 (1.45-2.23)	<0.001	1.35 (1.03-1.78)	0.03
**90 - <105**	1.02 (0.92-1.13)	0.69	1.18 (1.08-1.29)	<0.001	1.12 (0.99-1.26)	0.09
**75 - <90**	Ref	NA	Ref	NA	Ref	NA
**60 - <75**	1.08 (0.98-1.18)	0.12	1.08 (1.00-1.17)	0.06	1.01 (0.89-1.14)	0.86
**45 - <60**	1.21 (1.08-1.36)	0.001	1.15 (1.04-1.27)	0.01	0.95 (0.82-1.10)	0.49
**30 - <45**	1.08 (0.93-1.25)	0.31	1.11 (0.97-1.26)	0.13	1.02 (0.85-1.24)	0.81
**<30**	1.17 (0.92-1.50)	0.20	1.33 (1.09-1.62)	0.004	1.13 (0.83-1.53)	0.45

Logistic regression models adjusted for age, deprivation status, smoking status, comorbidity count and cancer site. eGFR: estimated glomerular filtration rate based on CKD-EPI 2009 equation and using serum creatinine. “Female” and “Male” models are stratified by sex. “Male versus female” model includes and interaction term between eGFR category and sex. Linear: P for linear trend. Non-linear: P for cubic trend. OR: odds ratio. CI: confidence interval.

Table S3 - Odds of presenting with advanced cancer by cancer site and eGFR category.

		Female		Male		Male versus female	
**Site**	**eGFR category (mL/min/1.73m^2)**	**OR (95% CI)**	**P value**	**OR (95% CI)**	**P value**	**OR (95% CI)**	**P value**
**Abdominal**	**P for trend**		Linear 0.94<br>Non-linear 0.03		Linear 0.15<br>Non-linear 0.20		Linear 0.13<br>Non-linear 0.01
**C22-26**	**>= 105**	0.39 (0.15-1.04)	0.06	2.27 (0.78-6.64)	0.13	6.13 (1.62-23.16)	0.01
	**90 - <105**	0.69 (0.42-1.14)	0.15	1.49 (0.91-2.45)	0.12	2.1 (1.1-4.03)	0.023
	**75 - <90**	Ref	NA	Ref	NA	Ref	NA
	**60 - <75**	0.89 (0.55-1.45)	0.64	0.92 (0.57-1.49)	0.73	0.98 (0.5-1.92)	0.95
	**45 - <60**	0.89 (0.49-1.62)	0.69	1.06 (0.57-1.98)	0.85	1.11 (0.48-2.57)	0.81
	**<45**	0.56 (0.31-1.04)	0.06	0.78 (0.4-1.54)	0.47	1.26 (0.53-2.98)	0.60
**Digestive tract**	**P for trend**		Linear 0.84<br>Non-linear 0.85		Linear 0.04<br>Non-linear 0.01		Linear 0.27<br>Non-linear 0.12
**C15-21**	**>= 105**	1.41 (0.79-2.5)	0.24	2.04 (1.29-3.21)	0.002	1.35 (0.67-2.71)	0.40
	**90 - <105**	1.04 (0.83-1.29)	0.76	1.18 (0.99-1.4)	0.06	1.11 (0.85-1.44)	0.45
	**75 - <90**	Ref	NA	Ref	NA	Ref	NA
	**60 - <75**	1.25 (1.04-1.51)	0.02	1.1 (0.94-1.28)	0.24	0.88 (0.69-1.12)	0.30
	**45 - <60**	1.3 (1.03-1.65)	0.03	1.09 (0.9-1.32)	0.39	0.84 (0.63-1.13)	0.25
	**<45**	0.96 (0.74-1.24)	0.75	0.98 (0.79-1.21)	0.83	1.03 (0.75-1.41)	0.87
**Head and neck**	**P for trend**		Linear 0.73<br>Non-linear 0.04		Linear 0.03<br>Non-linear 0.51		Linear 0.05<br>Non-linear 0.24
**C00-14**	**>= 105**	2.09 (0.81-5.44)	0.13	1.96 (1-3.84)	0.05	1.08 (0.38-3.04)	0.89
**C30-32**	**90 - <105**	2.08 (1.2-3.6)	0.01	1.24 (0.89-1.72)	0.21	0.59 (0.33-1.05)	0.07
	**75 - <90**	Ref	NA	Ref	NA	Ref	NA
	**60 - <75**	2.08 (1.1-3.95)	0.02	1.14 (0.79-1.66)	0.48	0.54 (0.26-1.11)	0.09
	**45 - <60**	0.86 (0.41-1.8)	0.69	1 (0.57-1.73)	0.99	1.07 (0.45-2.58)	0.87
	**<45**	2.2 (0.74-6.54)	0.16	0.57 (0.31-1.07)	0.08	0.25 (0.08-0.83)	0.02

**Lung**	**P for trend**		Linear 0.04<br>Non-linear 0.04		Linear 0.90<br>Non-linear 0.92		Linear 0.26<br>Non-linear 0.26
**C33-34**	**>= 105**	1.93 (1.03-3.61)	0.04	1.17 (0.7-1.96)	0.54	0.73 (0.33-1.59)	0.43
	**90 - <105**	1.17 (0.93-1.46)	0.18	1.09 (0.87-1.36)	0.47	1.01 (0.75-1.36)	0.95
	**75 - <90**	Ref	NA	Ref	NA	Ref	NA
	**60 - <75**	0.93 (0.76-1.14)	0.50	1.23 (1-1.52)	0.05	1.29 (0.96-1.73)	0.09
	**45 - <60**	0.99 (0.78-1.27)	0.95	1.14 (0.89-1.47)	0.29	1.09 (0.78-1.53)	0.60
	**<45**	0.9 (0.69-1.19)	0.47	1.02 (0.78-1.34)	0.87	1.06 (0.73-1.54)	0.75
**Melanoma**	**P for trend**		Linear 0.48<br>Non-linear 0.98		Linear 0.85<br>Non-linear 0.80		Linear 0.42<br>Non-linear 0.96
**C43**	**>= 105**	1.99 (0.55-7.24)	0.30	0.71 (0.18-2.86)	0.63	0.4 (0.08-2.05)	0.27
	**90 - <105**	1.57 (0.71-3.44)	0.26	0.56 (0.28-1.13)	0.11	0.35 (0.13-0.94)	0.04
	**75 - <90**	Ref	NA	Ref	NA	Ref	NA
	**60 - <75**	1.69 (0.84-3.43)	0.14	0.63 (0.35-1.13)	0.12	0.35 (0.14-0.87)	0.02
	**45 - <60**	1.4 (0.54-3.62)	0.48	0.84 (0.43-1.66)	0.62	0.61 (0.2-1.83)	0.38
	**<45**	0.64 (0.17-2.36)	0.50	1.01 (0.45-2.26)	0.98	1.66 (0.38-7.27)	0.50
**Other**	**P for trend**		Linear 0.61<br>Non-linear 0.46		Linear 0.40<br>Non-linear 0.12		Linear 0.58<br>Non-linear 0.28
**C37-38**	**>= 105**	8 (0.8-80.03)	0.08	3.04 (0.26-35.46)	0.38	1.24 (0.06-25.54)	0.89
**C45-49**	**90 - <105**	0.91 (0.27-3.04)	0.87	1.19 (0.54-2.6)	0.66	2.12 (0.58-7.72)	0.25
**C69-72**	**75 - <90**	Ref	NA	Ref	NA	Ref	NA
	**60 - <75**	0.71 (0.22-2.27)	0.56	0.87 (0.43-1.77)	0.70	1.36 (0.37-4.97)	0.65
	**45 - <60**	2.91 (0.52-16.28)	0.22	0.48 (0.19-1.21)	0.12	0.25 (0.04-1.44)	0.12
	**<45**	0.98 (0.17-5.76)	0.98	1.28 (0.34-4.81)	0.72	1.25 (0.16-9.9)	0.83
**Renal tract**	**P for trend**		Linear 0.17<br>Non-linear 0.44		Linear 0.03<br>Non-linear 0.33		Linear 0.85<br>Non-linear 0.75
**C64-67**	**>= 105**	1.05 (0.33-3.35)	0.94	0.81 (0.43-1.53)	0.52	0.82 (0.24-2.86)	0.75
	**90 - <105**	1.34 (0.81-2.2)	0.26	0.78 (0.55-1.12)	0.18	0.61 (0.34-1.09)	0.01

	**75 - <90**	Ref	NA	Ref	NA	Ref	NA
	**60 - <75**	1.56 (0.99-2.46)	0.06	1.04 (0.76-1.43)	0.81	0.66 (0.38-1.15)	0.15
	**45 - <60**	1.94 (1.24-3.03)	0.004	1.26 (0.92-1.74)	0.15	0.64 (0.38-1.09)	0.10
	**<45**	1.32 (0.81-2.13)	0.26	1.39 (0.99-1.95)	0.05	1.04 (0.6-1.8)	0.89
**Breast**	**P for trend**		Linear 0.01<br>Non-linear 0.06		NA		NA
**C50**	**>= 105**	1.65 (1.12-2.44)	0.01	NA	NA	NA	NA
	**90 - <105**	0.8 (0.65-0.99)	0.04	NA	NA	NA	NA
	**75 - <90**	Ref	NA	NA	NA	NA	NA
	**60 - <75**	1.08 (0.89-1.32)	0.43	NA	NA	NA	NA
	**45 - <60**	1.38 (1.07-1.79)	0.01	NA	NA	NA	NA
	**<45**	1.95 (1.44-2.63)	0	NA	NA	NA	NA
**Female genital tract**	**P for trend**		Linear 0.17<br>Non-linear 0.60		NA		NA
**C51-58**	**>= 105**	1 (0.62-1.61)	1.00	NA	NA	NA	NA
	**90 - <105**	0.93 (0.73-1.2)	0.59	NA	NA	NA	NA
	**75 - <90**	Ref	NA	NA	NA	NA	NA
	**60 - <75**	0.84 (0.67-1.06)	0.13	NA	NA	NA	NA
	**45 - <60**	1.16 (0.88-1.53)	0.29	NA	NA	NA	NA
	**<45**	1.41 (1.01-1.99)	0.05	NA	NA	NA	NA
**Prostate**	**P for trend**		NA		Linear 0.96<br>Non-linear <0.001		NA
**C61**	**>= 105**	NA	NA	2.02 (1.16-3.51)	0.01	NA	NA
	**90 - <105**	NA	NA	1.34 (1.16-1.56)	<0.001	NA	NA
	**75 - <90**	NA	NA	Ref	NA	NA	NA
	**60 - <75**	NA	NA	1.03 (0.9-1.17)	0.66	NA	NA
	**45 - <60**	NA	NA	1.13 (0.95-1.34)	0.17	NA	NA
	**<45**	NA	NA	1.48 (1.2-1.82)	<0.001	NA	NA

Logistic regression models adjusted for age, deprivation status, smoking status, comorbidity count and cancer site. “Female” and “Male” models are stratified by sex. “Male versus female” model includes and interaction term between eGFR category and sex. Linear: P for linear trend. Non-linear: P for cubic trend. eGFR: estimated glomerular filtration rate based on CKD-EPI 2009 equation and using serum creatinine. OR: odds ratio. CI: confidence interval.

Table S4 - Hazards of all-cause mortality across all sites by eGFR category.

	Female		Male		Male versus female	
**eGFR category (mL/min/1.73m^2)**	**HR (95% CI)**	**P value**	**HR (95% CI)**	**P value**	**HR (95% CI)**	**P value**
**P for trend**		Linear 0.16<br>Non-linear <0.001		Linear <0.001<br>Non-linear <0.001		Linear 0.27<br>Non-linear <0.001
**>120**	1.52 (0.93-2.5)	0.10	2.92 (2.21-3.85)	<0.001	1.8 (1.02-3.16)	0.04
**105 - <120**	1.63 (1.42-1.85)	<0.001	1.81 (1.63-2.02)	<0.001	1.06 (0.91-1.24)	0.46
**90 - <105**	1.17 (1.1-1.24)	<0.001	1.14 (1.08-1.2)	<0.001	0.95 (0.89-1.02)	0.17
**75 - <90**	Ref	NA	Ref	NA	Ref	NA
**60 - <75**	1.09 (1.03-1.14)	<0.001	1.03 (0.99-1.08)	0.13	0.96 (0.9-1.02)	0.17
**45 - <60**	1.19 (1.13-1.26)	<0.001	1.12 (1.07-1.18)	<0.001	0.96 (0.89-1.03)	0.24
**30 - <45**	1.3 (1.21-1.38)	<0.001	1.17 (1.11-1.25)	<0.001	0.93 (0.85-1.01)	0.07
**<30**	1.71 (1.56-1.88)	<0.001	1.48 (1.36-1.6)	<0.001	0.88 (0.78-0.99)	0.04

Cox proportional hazards models adjusted for age, deprivation status, smoking status, comorbidity count, cancer site and presenting cancer stage. eGFR: estimated glomerular filtration rate based on CKD-EPI 2009 equation and using serum creatinine. “Female” and “Male” models are stratified by sex. “Male versus female” model includes and interaction term between eGFR category and sex. Linear: P for linear trend. Non-linear: P for cubic trend. HR: hazards ratio. CI: confidence interval.

Table S5 – Age*eGFR interaction for hazards of all-cause mortality across all cancer sites.

Term	Female		Male	
	**HR (95% CI)**	**P value**	**HR (95% CI)**	**P value**
**Per 10 ml/min/1.73m^2 decrease in eGFR from reference group with age held constant**	1.23 (1.11-1.36)	<0.001	1.22 (1.10-1.35)	<0.001
**Additional effect of 10 ml/min/1.73m^2 decrease in eGFR per 10 years decreasing age**	1.02 (1.00-1.03)	0.01	1.02 (1.00-1.03)	0.01
**Per 10 ml/min/1.73m^2 increase in eGFR from reference group with age held constant**	1.35 (1.17-1.54)	<0.001	1.66 (1.46-1.89)	<0.001
**Additional effect of 10 ml/min/1.73m^2 increase in eGFR per 10 years decreasing age**	1.02 (1.00-1.05)	0.04	1.05 (1.03-1.08)	<0.001

Cox proportional hazards models adjusted for age, deprivation status, smoking status, comorbidity count, cancer site and presenting cancer stage. eGFR: estimated glomerular filtration rate based on CKD-EPI 2009 equation and using serum creatinine. “Female” and “Male” models are stratified by sex. HR: hazards ratio. CI: confidence interval.

Table S6 - Hazards of all-cause mortality by cancer site and eGFR category.

		Female		Male		Male versus female	
**Site**	**eGFR category (mL/min/1.73m^2)**	**HR (95% CI)**	**P value**	**HR (95% CI)**	**P value**	**HR (95% CI)**	**P value**
**Abdominal**	**P for trend**		Linear 0.48<br>Non-linear 0.02		Linear 0.91<br>Non-linear 0.02		Linear 0.48<br>Non-linear 0.17
**C22-26**	**>= 105**	1.67 (1.12-2.48)	0.01	1.26 (0.96-1.66)	0.10	0.78 (0.5-1.22)	0.27
	**90 - <105**	1.09 (0.91-1.31)	0.33	1.04 (0.9-1.2)	0.63	0.97 (0.79-1.19)	0.76
	**75 - <90**	Ref	NA	Ref	NA	Ref	NA
	**60 - <75**	1.08 (0.94-1.25)	0.29	1.06 (0.93-1.22)	0.38	0.99 (0.81-1.2)	0.88
	**45 - <60**	1.22 (1.03-1.44)	0.02	1.13 (0.95-1.33)	0.16	0.92 (0.73-1.15)	0.47
	**<45**	1.37 (1.15-1.64)	<0.001	1.08 (0.91-1.3)	0.38	0.78 (0.61-0.99)	0.04
**Digestive tract**	**P for trend**		Linear 0.87<br>Non-linear <0.001		Linear 0.04<br>Non-linear <0.001		Linear 0.03<br>Non-linear 0.47
**C15-21**	**>= 105**	1.91 (1.4-2.6)	<0.001	2.2 (1.77-2.73)	0	1.18 (0.83-1.68)	0.35
	**90 - <105**	1.14 (1-1.31)	0.05	1.11 (1.01-1.23)	0.03	1 (0.85-1.17)	0.96
	**75 - <90**	Ref	NA	Ref	NA	Ref	NA
	**60 - <75**	1.11 (1-1.23)	0.04	1.01 (0.93-1.1)	0.79	0.89 (0.78-1.02)	0.09
	**45 - <60**	1.14 (1.01-1.29)	0.03	1.14 (1.04-1.26)	0.006	0.97 (0.84-1.13)	0.71
	**<45**	1.4 (1.23-1.59)	<0.001	1.23 (1.11-1.37)	<0.001	0.85 (0.73-1)	0.04
**Head and neck**	**P for trend**		Linear 0.26<br>Non-linear 0.77		Linear 0.16<br>Non-linear 0.01		Linear 0.43<br>Non-linear 0.53
**C00-14**	**>= 105**	1.69 (0.88-3.24)	0.11	1.98 (1.4-2.82)	<0.001	0.89 (0.47-1.69)	0.73
**C30-32**	**90 - <105**	1.18 (0.82-1.7)	0.37	1.16 (0.95-1.42)	0.14	0.84 (0.57-1.22)	0.36
	**75 - <90**	Ref	NA	Ref	NA	Ref	NA
	**60 - <75**	1.22 (0.84-1.77)	0.29	1 (0.8-1.24)	0.98	0.85 (0.55-1.29)	0.44
	**45 - <60**	1.93 (1.27-2.93)	0.002	1.13 (0.84-1.51)	0.43	0.63 (0.39-1.02)	0.06
	**<45**	1.46 (0.84-2.55)	0.18	1.02 (0.72-1.45)	0.89	0.91 (0.49-1.67)	0.76

**Lung**	**P for trend**		Linear 0.94<br>Non-linear <0.001		Linear 0.19<br>Non-linear <0.001		Linear 0.35<br>Non-linear 0.54
**C33-34**	**>= 105**	1.67 (1.32-2.13)	<0.001	1.52 (1.24-1.86)	0	0.93 (0.69-1.25)	0.61
	**90 - <105**	1.18 (1.07-1.3)	<0.001	1.13 (1.03-1.24)	0.008	0.97 (0.85-1.1)	0.62
	**75 - <90**	Ref	NA	Ref	NA	Ref	NA
	**60 - <75**	1.09 (1-1.2)	0.05	1 (0.92-1.09)	0.99	0.92 (0.81-1.04)	0.19
	**45 - <60**	1.14 (1.02-1.26)	0.02	1.09 (0.99-1.21)	0.09	0.96 (0.84-1.11)	0.61
	**<45**	1.27 (1.13-1.43)	<0.001	1.1 (0.99-1.23)	0.09	0.88 (0.75-1.03)	0.10
**Melanoma**	**P for trend**		Linear 0.37<br>Non-linear 0.04		Linear 0.88<br>Non-linear 0.002		Linear 0.98<br>Non-linear 0.61
**C43**	**>= 105**	3.35 (1.36-8.25)	0.01	3.5 (1.51-8.12)	0.004	1.04 (0.36-3.04)	0.94
	**90 - <105**	1.26 (0.74-2.13)	0.40	1.74 (1.19-2.55)	0.004	1.32 (0.73-2.41)	0.36
	**75 - <90**	Ref	NA	Ref	NA	Ref	NA
	**60 - <75**	1.2 (0.83-1.73)	0.34	1.35 (1-1.82)	0.05	1.17 (0.73-1.86)	0.52
	**45 - <60**	1.25 (0.84-1.88)	0.27	1.02 (0.72-1.43)	0.93	0.89 (0.54-1.47)	0.66
	**<45**	1.25 (0.79-1.96)	0.34	1.18 (0.82-1.71)	0.38	1.14 (0.65-1.98)	0.65
**Other**	**P for trend**		Linear 0.71<br>Non-linear <0.001		Linear 0.03<br>Non-linear 0.82		Linear 0.37<br>Non-linear 0.05
**C37-38**	**>= 105**	1.69 (0.95-3.01)	0.08	0.72 (0.43-1.22)	0.23	0.67 (0.33-1.34)	0.25
**C45-49**	**90 - <105**	1.17 (0.88-1.55)	0.28	0.95 (0.78-1.17)	0.65	0.99 (0.72-1.36)	0.94
**C69-72**	**75 - <90**	Ref	NA	Ref	NA	Ref	NA
	**60 - <75**	0.85 (0.66-1.09)	0.19	0.95 (0.8-1.14)	0.59	1.07 (0.79-1.45)	0.66
	**45 - <60**	0.81 (0.59-1.11)	0.20	0.99 (0.79-1.24)	0.92	0.99 (0.7-1.42)	0.97
	**<45**	1.86 (1.3-2.64)	<0.001	1.4 (1.08-1.81)	0.01	0.59 (0.4-0.88)	0.009
**Renal tract**	**P for trend**		Linear 0.24<br>Non-linear 0.68		Linear 0.01<br>Non-linear 0.95		Linear 0.70<br>Non-linear 0.54
**C64-67**	**>= 105**	0.72 (0.23-2.3)	0.58	1.03 (0.64-1.66)	0.91	1.58 (0.46-5.41)	0.47
	**90 - <105**	1.03 (0.75-1.42)	0.84	0.9 (0.72-1.13)	0.37	0.94 (0.65-1.36)	0.73

	**75 - <90**	Ref	NA	Ref	NA	Ref	NA
	**60 - <75**	1.08 (0.85-1.37)	0.53	1.09 (0.92-1.28)	0.33	0.99 (0.74-1.33)	0.96
	**45 - <60**	1.06 (0.83-1.34)	0.66	1.17 (0.99-1.37)	0.06	1.08 (0.81-1.43)	0.60
	**<45**	1.25 (0.99-1.58)	0.06	1.41 (1.2-1.66)	<0.001	1.06 (0.82-1.38)	0.65
**Breast**	**P for trend**		Linear 0.37<br>Non-linear 0.002		NA		NA
**C50**	**>= 105**	1.82 (1.27-2.59)	<0.001	NA	NA	NA	NA
	**90 - <105**	1.17 (0.99-1.37)	0.06	NA	NA	NA	NA
	**75 - <90**	Ref	NA	NA	NA	NA	NA
	**60 - <75**	1.13 (0.99-1.28)	0.07	NA	NA	NA	NA
	**45 - <60**	1.26 (1.09-1.46)	0.002	NA	NA	NA	NA
	**<45**	1.44 (1.24-1.68)	<0.001	NA	NA	NA	NA
**Female genital tract**	**P for trend**		Linear 0.001<br>Non-linear 0.43		NA		NA
**C51-58**	**>= 105**	1.07 (0.71-1.61)	0.74	NA	NA	NA	NA
	**90 - <105**	1.16 (0.98-1.38)	0.09	NA	NA	NA	NA
	**75 - <90**	Ref	NA	NA	NA	NA	NA
	**60 - <75**	1.03 (0.89-1.18)	0.71	NA	NA	NA	NA
	**45 - <60**	1.35 (1.15-1.57)	<0.001	NA	NA	NA	NA
	**<45**	1.61 (1.36-1.91)	<0.001	NA	NA	NA	NA
**Prostate**	**P for trend**		NA		Linear 0.79<br>Non-linear 0.002		NA
**C61**	**>= 105**	NA	NA	1.86 (1.11-3.12)	0.02	NA	NA
	**90 - <105**	NA	NA	1.24 (1.08-1.42)	0.003	NA	NA
	**75 - <90**	NA	NA	Ref	NA	NA	NA
	**60 - <75**	NA	NA	1.03 (0.93-1.14)	0.55	NA	NA
	**45 - <60**	NA	NA	1.09 (0.97-1.23)	0.14	NA	NA
	**<45**	NA	NA	1.36 (1.21-1.53)	<0.001	NA	NA

Cox proportional hazards models adjusted for age, deprivation status, smoking status, comorbidity count, cancer site and presenting cancer stage. eGFR: estimated glomerular filtration rate based on CKD-EPI 2009 equation and using serum creatinine. “Female” and “Male” models are stratified by sex. “Male versus female” model includes and interaction term between eGFR category and sex. Linear: P for linear trend. Non-linear: P for cubic trend. HR: hazards ratio. CI: confidence interval.

Figure S1

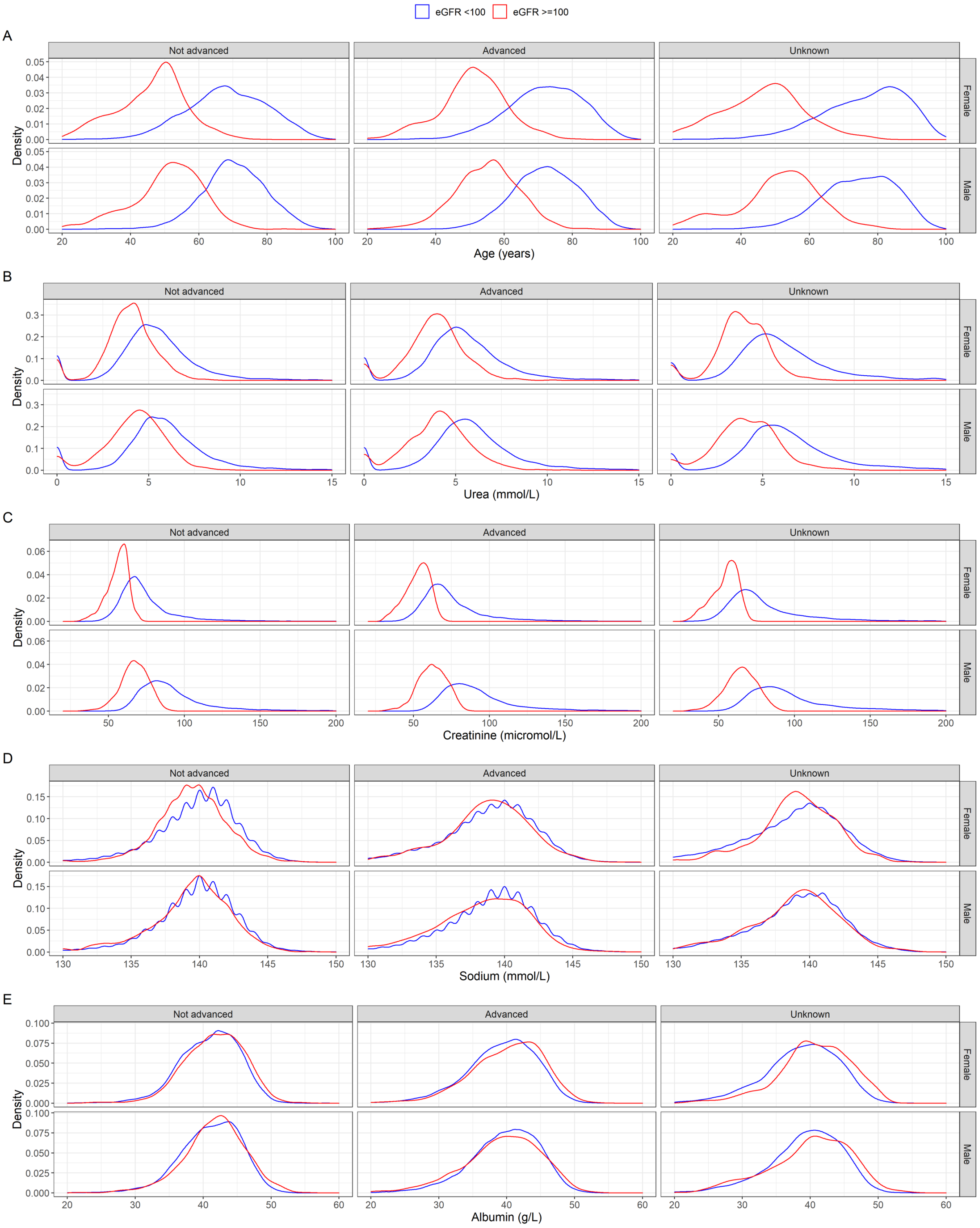

Density plots illustration the distribution of age, serum urea, creatinine, sodium and albumin according to sex, cancer stage at diagnosis and eGFR.

Figure S2

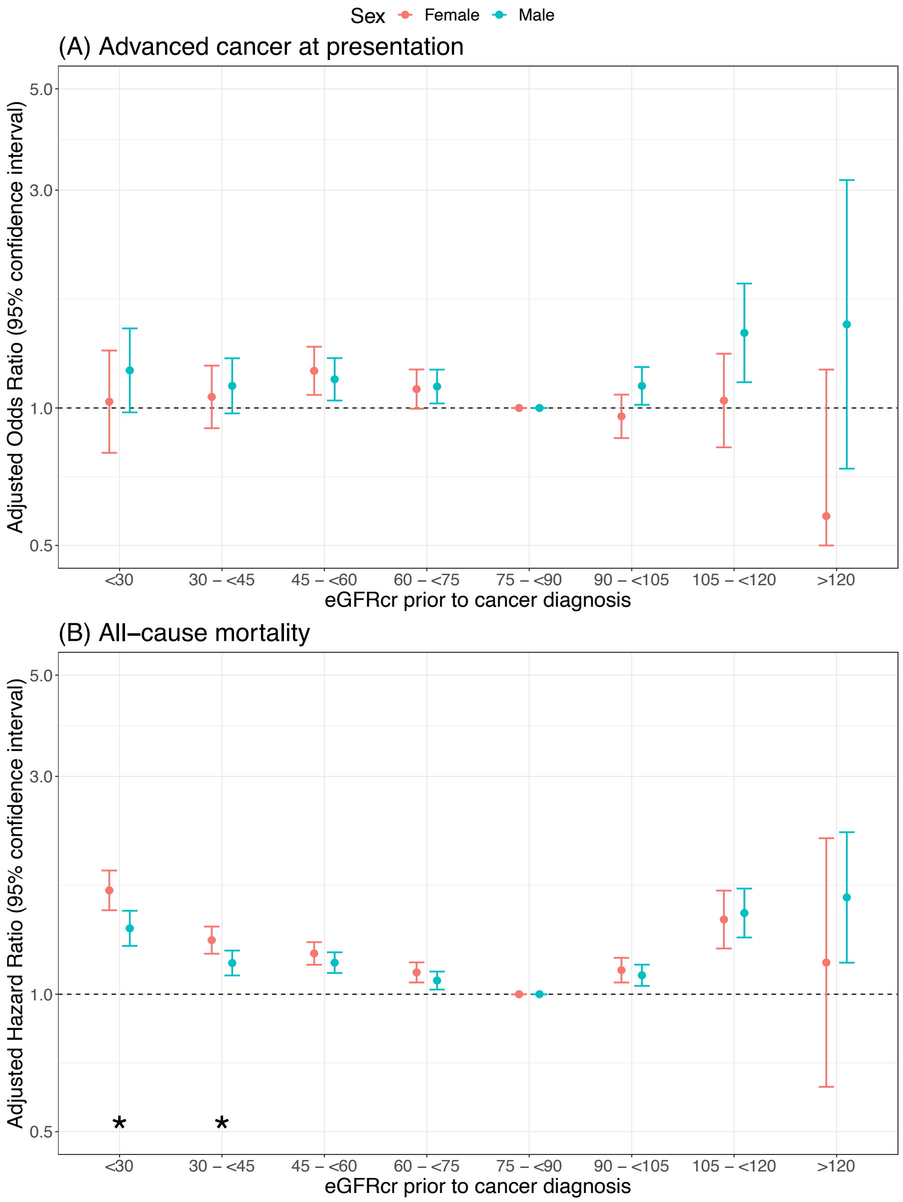

(A) Plot displaying OR (95% CI) of presentation with advanced cancer, adjusted for age, smoking status, deprivation status, number of comorbidities, cancer site, urea, sodium and albumin at baseline.

(B) Plot displaying HR (95% CI) of death after cancer diagnosis, adjusted for age, smoking status, deprivation status, number of comorbidities, cancer site, presenting cancer stage, urea, sodium and albumin at baseline.

Results are stratified by sex. * indicates presence of a significant interaction between sex and eGFR category. Reference eGFR category: 75 - <90 mL/min/1.73m^2.
